# Simple Monitoring of Gene Targeting Efficiency in Human Somatic Cell Lines Using the *PIGA* Gene

**DOI:** 10.1371/journal.pone.0047389

**Published:** 2012-10-08

**Authors:** Sivasundaram Karnan, Yuko Konishi, Akinobu Ota, Miyuki Takahashi, Lkhagvasuren Damdindorj, Yoshitaka Hosokawa, Hiroyuki Konishi

**Affiliations:** Department of Biochemistry, Aichi Medical University School of Medicine, Nagakute, Aichi, Japan; University of Kansas Medical Center, United States of America

## Abstract

Gene targeting in most of human somatic cell lines has been labor-intensive because of low homologous recombination efficiency. The development of an experimental system that permits a facile evaluation of gene targeting efficiency in human somatic cell lines is the first step towards the improvement of this technology and its application to a broad range of cell lines. In this study, we utilized phosphatidylinositol glycan anchor biosynthesis class A (*PIGA*), a gene essential for the synthesis of glycosylphosphatidyl inositol (GPI) anchors, as a reporter of gene targeting events in human somatic cell lines. Targeted disruption of *PIGA* was quantitatively detected with FLAER, a reagent that specifically binds to GPI anchors. Using this *PIGA*-based reporter system, we successfully detected adeno-associated virus (AAV)-mediated gene targeting events both with and without promoter-trap enrichment of gene-targeted cell population. The *PIGA*-based reporter system was also capable of reproducing previous findings that an AAV-mediated gene targeting achieves a remarkably higher ratio of homologous versus random integration (H/R ratio) of targeting vectors than a plasmid-mediated gene targeting. The *PIGA*-based system also detected an approximately 2-fold increase in the H/R ratio achieved by a small negative selection cassette introduced at the end of the AAV-based targeting vector with a promoter-trap system. Thus, our *PIGA*-based system is useful for monitoring AAV-mediated gene targeting and will assist in improving gene targeting technology in human somatic cell lines.

## Introduction

Gene targeting is a powerful technology to explore gene functions in eukaryotes by allowing the manipulation of endogenous genes via homologous recombination (HR). Although gene targeting in mammalian cells has been mainly conducted in murine embryonic stem (ES) cells for the purpose of creating knockout or knock-in mouse models, human somatic cells have also been subjected to gene targeting [1]. Gene-targeted human cell lines are useful to study human gene functions and diseases caused by genetic alterations, such as cancer [2], [3]. These gene-targeted cell lines are also believed to be promising drug screening platforms used for the development and validation of molecularly targeted therapies [4], [5].

Several significant improvements in gene targeting technology have been made in previous studies, including the use of adeno-associated virus (AAV)-based targeting vectors [6], [7], a promoter-trap strategy with the aid of internal ribosomal entry sites (IRES) [8], and a systematic PCR screening method for gene-targeted cell clones [9]. Despite these improvements, gene targeting in most of human somatic cell lines remains to be difficult probably because of low HR efficiencies as demonstrated in murine somatic cells [10]. The vast majority of targeting vectors introduced into human somatic cells are integrated randomly, rather than via HR, into the genome. To further improve this technology and make it readily applicable to a wide range of human cell lines, it is advantageous to have an experimental system monitoring gene targeting efficiencies with a simple procedure. In particular, monitoring the frequency of homologous versus random integration (H/R ratio) of targeting vectors is informative, as this ratio is a major determinant of difficulty in conducting gene targeting.

The phosphatidylinositol glycan anchor biosynthesis class A (*PIGA*) gene on the X chromosome encodes a protein that is required for glycosylphosphatidyl inositol (GPI) anchor biosynthesis [11], [12]. An inactivating mutation in the *PIGA* gene results in the loss of GPI anchors in the cells carrying a single X chromosome, such as diploid cells of male origin. The loss of GPI anchors then leads to the release of extracellular proteins that are otherwise linked to cell membranes by GPI anchors. Cells without GPI anchors are readily detectable with negative staining by reagents that bind to GPI anchors or by antibodies targeted against GPI-anchored proteins [13], [14]. Thus, the frequency of *PIGA* inactivation can be monitored by simple cell staining followed by fluorescence flow cytometry. This unique property of *PIGA* has been exploited to estimate the incidence of genetic mutations occurring spontaneously or elicited by mutagens during cell culture or in animal models [15]–[18]. *PIGA* was also disrupted by zinc-finger nucleases (ZFNs) in human ES cells and induced pluripotent stem cells to provide a proof of principle for the genome editing mediated by ZFNs [19]. In the current study, we used *PIGA* to develop an assay that evaluates gene targeting efficiencies represented by H/R ratios in human somatic cell lines. Using this system, we also attempted to modify the structure of an AAV-based targeting vector and thereby improve the gene targeting efficiency in order to facilitate the application of this technology to various human somatic cell lines.

## Results

### Quantitative measurement of *PIGA* gene targeting frequency

**Figure 1:**
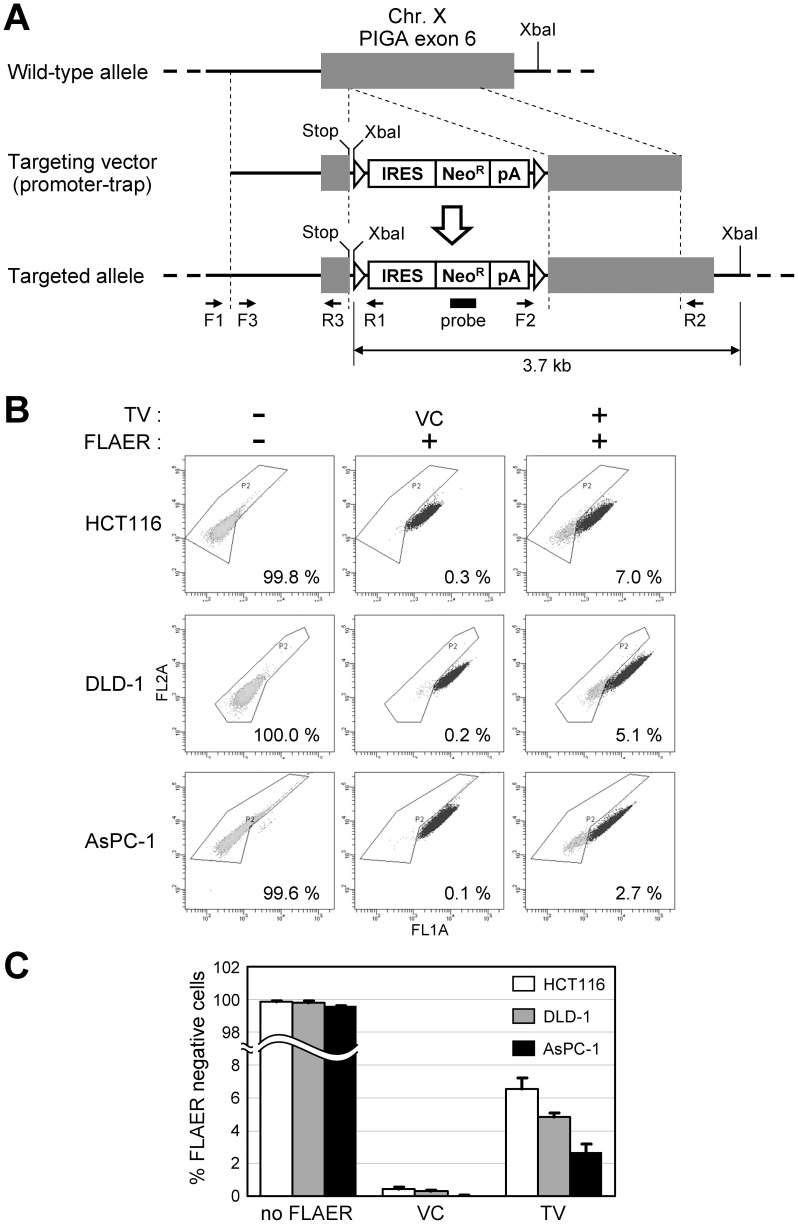
*PIGA* gene targeting in human somatic cell lines. (A) A diagram of the promoter-trap *PIGA* targeting vector. Gray boxes indicate *PIGA* exon 6, while thick lines indicate *PIGA* intron 5 and intergenic genomic region. Thin dotted lines indicate homology between the endogenous gene locus and the targeting vector. F1 to F3 and R1 to R3 indicate PCR primers used in the experiment shown in Fig. 2A and B. A filled rectangle indicates the location of the probe used in Southern blot analysis shown in Fig. 2C. XbaI restriction enzyme sites are marked in the diagram. An arrow at the bottom shows the distance between two XbaI sites at the targeted *PIGA* gene locus. Triangles: loxP; Stop: stop codon (TAA); IRES: internal ribosomal entry site; Neo^R^: neomycin resistance gene; pA: polyadenylation site. (B and C) Human somatic cell lines were infected with the promoter-trap *PIGA* targeting vector or a vector control (VC), and processed for FLAER staining and then flow cytometric analyses. Shown are the representative dot plots (B) and a graphic representation of the entire results (mean ± s.e.m.; *n* = 3) (C). In (B), the FLAER-negative ratio for each experiment is noted in the dot plot. TV: promoter-trap *PIGA* targeting vector.

We initially constructed a targeting vector that disrupts *PIGA* exon 6 as shown in Fig. 1A. This targeting vector has 5′ and 3′ homology arms, both of which are approximately 1 kb in length. This targeting vector also has a neomycin resistance gene (Neo^R^) downstream of an IRES that allows for the enrichment of gene-targeted clones by promoter-trap strategy. In addition, the vector has two loxP sites encompassing the Neo^R^ expression cassette so that Neo^R^ can be excised by Cre-loxP recombination, which allows Neo^R^ to be reused for further genetic manipulation. An artificial stop codon within exon 6 was incorporated at the proximal end of the 5′ arm to ensure *PIGA* disruption. This targeting vector was created based on an AAV backbone, as AAV-based targeting vectors reportedly achieve more than 1,000-times higher gene targeting frequencies than conventional plasmid-based targeting vectors [6], and have been employed in many recent studies to overcome low gene targeting efficiencies in human somatic cell lines [5], [20]–[28].

**Figure 2:**
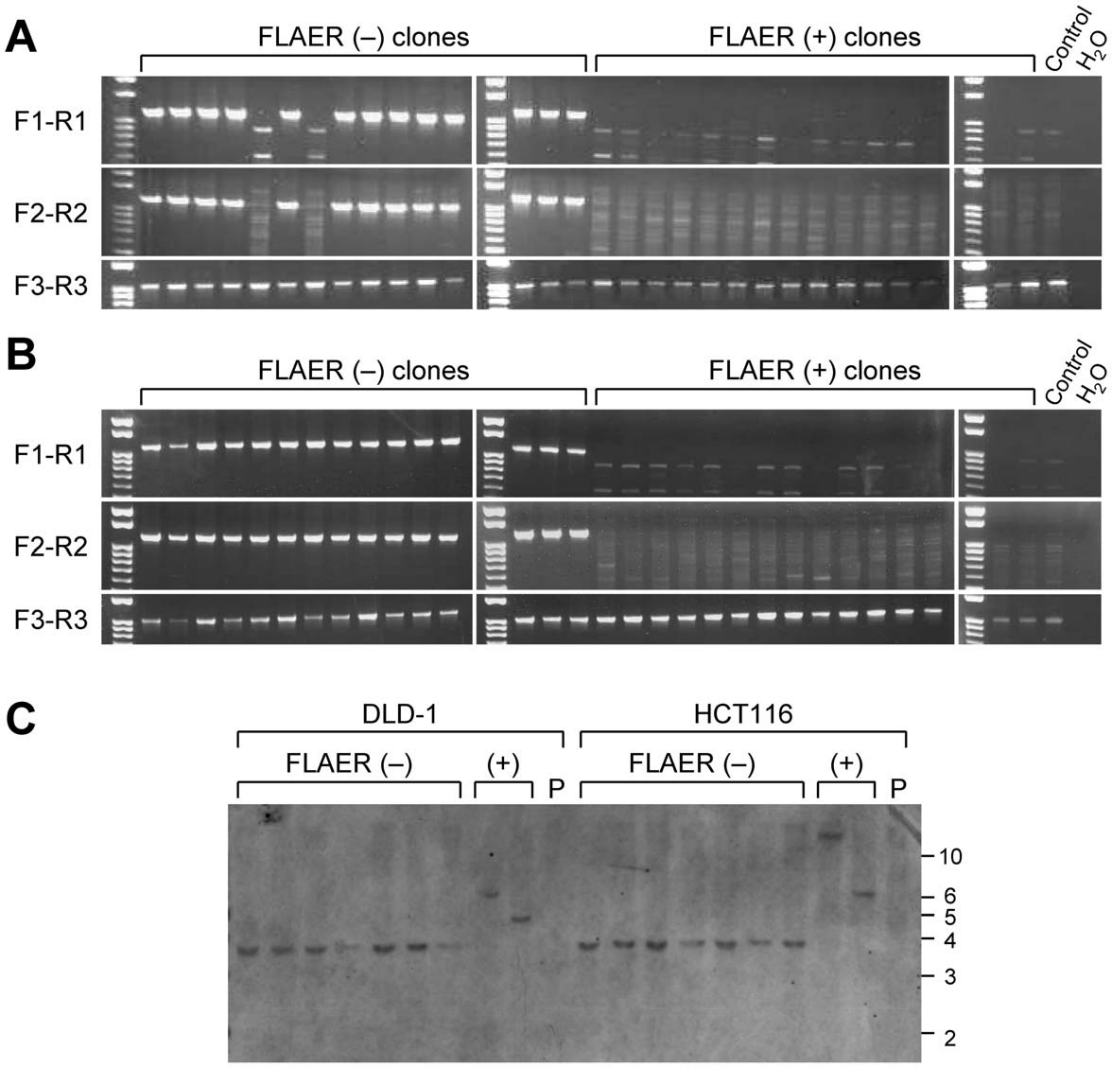
Confirmatory assays examining the *PIGA* gene locus in FLAER-negative and -positive cell clones. (A and B) Representative results of PCR examining FLAER-negative and -positive single cell clones derived from HCT116 (A) and DLD-1 (B) infected with the promoter-trap *PIGA* targeting vector. PCR was conducted with primer pairs F1-R1, F2-R2 and F3-R3 that yielded PCR products of 1,140 bp, 1,184 bp, and 1,031 bp in size, respectively. For the location of primers, see Fig. 1A. Control: parental cell lines. (C) Southern blot analysis of FLAER-negative and -positive single cell clones. Genomic DNA was digested with XbaI, separated on a gel, and hybridized with a probe shown in Fig. 1A. The positions of the DNA size standards are shown to the right in kilobase. P: parental cell lines.

The constructed AAV-based *PIGA* targeting vector was used to infect two colon cancer cell lines HCT116 and DLD-1 and a pancreatic cancer cell line AsPC-1, all of which are of male origin. The infected cells were selected with G418 to assess the H/R ratio of the targeting vector, and stained with an Alexa 488-conjugated inactive aerolysin variant, FLAER, which specifically binds to GPI-anchors [29]. Cells were then analyzed by fluorescence flow cytometry to determine the percentage of FLAER-negative cells. As expected, virtually all the cells not processed for FLAER staining did not have an Alexa 488 signal, whereas the cells that were processed for FLAER staining but that had been infected with an unrelated AAV vector driving Neo^R^ gene expression (vector control; VC) were mostly FLAER-positive (Fig. 1B and C). In contrast, FLAER-negative populations accounted for 2.7–7.0% of the cells that were subjected to both *PIGA* gene targeting and FLAER staining, suggesting that gene targeting successfully inactivated *PIGA*. We subsequently sorted both FLAER-negative and -positive populations from HCT116 and DLD-1 cells infected with the *PIGA* targeting vector, and established 55 and 15 single cell clones from FLAER-negative and -positive populations, respectively, for both cell lines. The resulting cell clones were examined with a pair of analytical PCR reactions encompassing the 5′ and 3′ homology arms of the targeting vector, respectively. These PCR reactions demonstrated that 51 of 55 (93%; HCT116) and 54 of 55 (98%; DLD-1) FLAER-negative clones underwent *PIGA* gene targeting, while all 15 FLAER-positive clones were apparently formed by the random integration of the targeting vector into the genome (Fig. 2A and B). We also confirmed the DNA sequence around the targeted genomic locus at the *PIGA* exon 6 in 15 DLD-1-derived FLAER-negative cell clones (data not shown). We further analyzed a subset of FLAER-negative and -positive cell clones derived from HCT116 and DLD-1 by Southern blotting, and thereby confirmed that the *PIGA* gene had been targeted as expected in FLAER-negative cell clones (Fig. 2C). These data collectively indicate that the negative FLAER staining in our system truly represents the abrogation of *PIGA* by gene targeting.

### Disruption of *PIGA* does not affect the proliferation of HCT116 and DLD-1 cells

**Figure 3:**
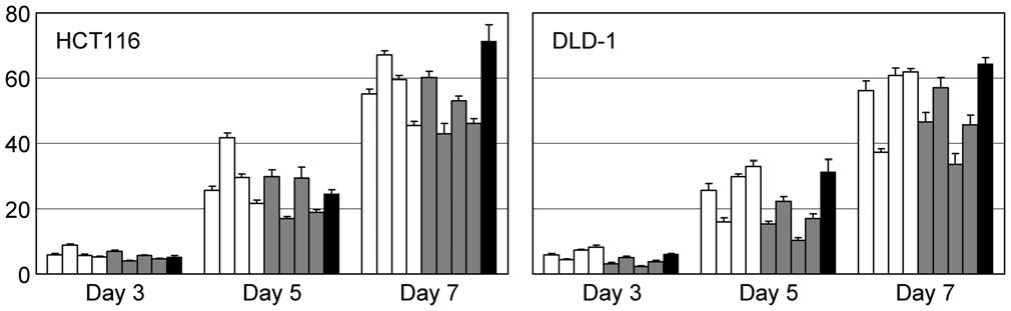
Growth indices of *PIGA*-targeted and non-targeted cell populations. Cells derived from HCT116 (left) and DLD-1 (right) were propagated in 96-well tissue culture plates, and cell numbers assayed at Day 3, Day 5, and Day 7 were shown relative to those at Day 1. In each cluster of bar graphs, open bars represent a FLAER-negative bulk cell population and *PIGA*-targeted (confirmed by Southern blotting) single cell clones #1, #2 and #3 (from left to right); gray bars represent a FLAER-positive bulk cell population and *PIGA*-non-targeted (determined by analytical PCR) single cell clones #1, #2 and #3 (from left to right); a filled bar represents the parental cell line (mean ± s.d.; *n* = 6).

*PIGA* encodes a protein with important cellular function. If the disruption of *PIGA* affects cell proliferation, flow cytometric analysis of the cells transduced with *PIGA* targeting vector after a period of antibiotic selection may incorrectly estimate gene targeting efficiency. To clarify whether *PIGA*-deficient cells have growth advantage or disadvantage against *PIGA*-proficient cells, we next compared the growth indices of FLAER-negative bulk cell populations and single cell clones derived from them with those of the FLAER-positive bulk cell populations, single cell clones derived from them, and parental cell lines. As shown in Fig. 3, no significant difference in cell growth was observed between *PIGA*-deficient and -proficient cells both in HCT116 and DLD-1 series.

### Gene targeting in human somatic cell lines with various experimental conditions

**Figure 4:**
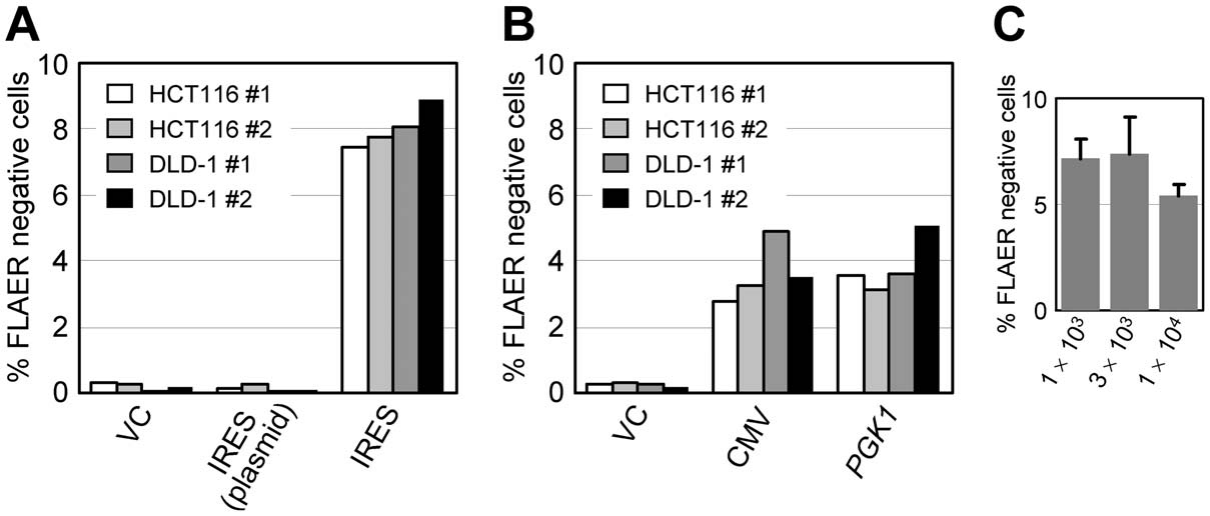
Monitoring of the gene targeting efficiencies in various experimental conditions using the *PIGA*-based reporter system. (A) HCT116 and DLD-1 cells were transfected (as a plasmid) or infected (as an AAV) with the promoter-trap *PIGA* targeting vector, and the ratios of FLAER-negative cell populations were determined in duplicate. In X-axis labels, VC, IRES (plasmid) and IRES indicate a vector control, and the plasmid and AAV versions of promoter-trap *PIGA* targeting vector, respectively. (B) HCT116 and DLD-1 cells were infected with *PIGA* targeting vectors in which the Neo^R^ gene was driven by the cytomegalovirus (CMV) promoter or the promoter of human phosphoglycerate kinase 1 (*PGK1*) gene, and the ratios of FLAER-negative cell populations were determined in duplicate. (C) HCT116 was infected with the indicated multiplicity of infection of the AAV-based promoter-trap *PIGA* targeting vector, and the ratios of FLAER-negative cell populations were determined (mean ± s.e.m.; *n* = 3).

We subsequently investigated whether our *PIGA*-based system can reproduce a previous finding that an AAV-based targeting vector achieves several orders of magnitude greater targeting efficiency than a plasmid-based targeting vector that, except for its backbone, has an identical structure [6]. Since HCT116 and DLD-1 were found to be more amenable to *PIGA* gene targeting than AsPC-1 in our system (Fig. 1B and C), we used these two cell lines in most of the experiments hereafter performed. We first transfected (as a plasmid) or infected (as an AAV) our *PIGA* targeting vector into the human cell lines, selected the cells with G418, and then compared the percentages of FLAER-negative cells between the plasmid and AAV groups. We detected no increase of FLAER-negative cells by the transfection of *PIGA* targeting vector; however, the infection of the cells with *PIGA* targeting vector resulted in an increase of FLAER-negative cell populations to 7.4–8.9% (*p* <0.0001, versus the plasmid-based targeting vector; Fig. 4A). These data suggest that, in the *PIGA* gene targeting, an AAV-based targeting vector promotes a higher gene targeting frequency than a plasmid-based vector consistent with previous studies [6], [8], [30]. However, our *PIGA*-based system may not be sufficiently sensitive to detect low gene targeting frequencies achieved by plasmid-based targeting vectors in human somatic cell lines.

We next investigated whether our *PIGA*-based system can detect AAV-mediated gene targeting performed without a promoter-trap system. To this end, we created a pair of new *PIGA* targeting vectors by replacing IRES within the above-described promoter-trap *PIGA* targeting vector with either the cytomegalovirus (CMV) immediate early promoter or the promoter of human phosphoglycerate kinase 1 (*PGK1*) gene. AAV was produced with these constructs and then used for the infection of the HCT116 and DLD-1 cell lines. Flow cytometric analysis of the cell lines after G418 selection exhibited an increase of FLAER-negative cell populations to 2.8–5.1% (*p* = 0.0005 for CMV versus VC, and *p* = 0.0004 for *PGK1* versus VC; Fig. 4B), suggesting that our *PIGA*-based system is capable of detecting gene targeting events elicited by AAV-based non-promoter-trap targeting vectors (Fig. 4B).

We also examined whether the multiplicity of infection (MOI) of the *PIGA* targeting vector has an impact on the H/R ratio in our system. To this end, HCT116 cells were infected with the promoter-trap *PIGA* targeting vector at MOIs between 1×10^3^ and 1×10^4^, and processed for the quantification of FLAER-negative cell populations as described above. As a result, no significant change in the H/R ratio was observed within this range of MOIs (Fig. 4C). It has been shown that both homologous and random integration of AAV-based targeting vectors increase as the MOI increases [6], [31], [32]. A previous study also showed that, unlike our results, the H/R ratio of an AAV-based targeting vector increases in an MOI-dependent manner [32], even though this increase may be compromised because of the counterbalance between two parameters, *i.e.*, homologous and random integration. The inconsistency between the results of this study and ours may be due to the different experimental systems that were used to determine gene targeting frequencies (GFP-based versus *PIGA*-based systems) or the different types of cells employed for gene targeting (293 cells versus colon cancer cell lines). However, a possibility remains that limited sensitivity in determining H/R ratio in our system might account for the absence of the correlation between MOI and H/R ratio in our experimental results.

### Positive-negative selection (PNS) in an AAV-based targeting vector

**Figure 5:**
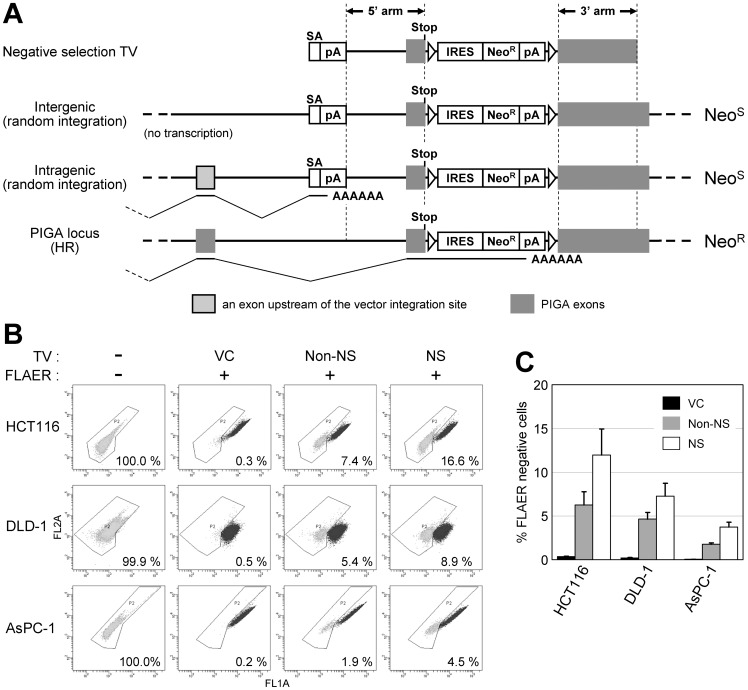
Negative selection (NS) in AAV-mediated gene targeting. (A) A diagram describing the NS conferred by a small cassette consisting of splice acceptor (SA) and a polyadenylation site (pA). “Negative selection TV” depicts a promoter-trap *PIGA* targeting vector with a NS cassette. Other notations on the left indicate the location and the manner (in parenthesis) of vector integration. Bold and thin lines depict introns and anticipated splicing in the locus, respectively. Noted to the right are the theoretical neomycin sensitive (Neo^S^) or resistant (Neo^R^) statuses of the cells resulting from the integration of vectors. HR: homologous recombination. (B and C) Cell lines were infected with indicated *PIGA* targeting vectors or a vector control (VC), and processed for FLAER staining and then flow cytometric analyses. Shown are the representative dot plots (B) and a graphic representation of the entire results (mean ± s.e.m.; *n* = 3) (C). In (B), the FLAER-negative ratio for each experiment is noted in the dot plot. Non-NS: a promoter-trap *PIGA* targeting vector without the NS cassette (depicted in Fig. 1A).

We next utilized this *PIGA*-based reporter system in an attempt to employ PNS in the context of AAV-mediated gene targeting. PNS is a useful strategy to enrich gene-targeted cell populations during plasmid-based gene targeting [33] and has been primarily used in murine ES cells. Negative selection (NS) is accomplished by inserting a genetic cassette that contains a gene conferring cytotoxicity or drug sensitivity, such as herpes simplex virus thymidine kinase [33] and diphtheria toxin A fragment [34], at the distal end of either of the homology arms. To the best of our knowledge, PNS has not been utilized in AAV-based targeting vectors because AAV vectors have a strict size limit of approximately 5 kb in total, and NS cassettes described above are too large to be used in AAV-based targeting vectors. Thus, in place of a large gene for NS, we tested a small cassette comprising a splice acceptor derived from human *BCL2* intron 2/exon 3 boundary followed by a bovine growth hormone (BGH) polyadenylation site (polyA) inserted at the distal end of the 5′ arm in the *PIGA* targeting vector (Fig. 5A). This cassette is 524 bp long in total and is thus sufficiently small to place within an AAV vector. When a targeting vector carrying this cassette, along with an IRES-based promoter-trap system, is randomly integrated into an intragenic region of the genome, this cassette will trap at least a part of transcription machinery at the disrupted endogenous gene locus, and thereby reduce Neo^R^ gene expression. In contrast, when the targeting vector is integrated into the *PIGA* gene locus via HR, this cassette will be removed upon integration, and thus the Neo^R^ gene will be transcribed by the endogenous *PIGA* promoter without interruption. Thus, this small cassette in principle will provide NS when used with a promoter-trap system. To our knowledge, this is the first report officially assessing the impact of this type of NS cassette on gene targeting efficiency.

We assayed the difference in gene targeting efficiencies achieved by a promoter-trap *PIGA* targeting vector between in the presence and absence of this NS cassette in the vector. HCT116, DLD-1, and AsPC-1 cells were infected with these AAV-based targeting vectors, selected with G418, stained with FLAER, and then processed for flow cytometry to quantify FLAER-negative cells. These assays demonstrated a 1.6- to 2.1-fold enrichment of gene-targeted clones by the use of the NS cassette (*p*<0.05; Fig. 5B and C). In accord with this enrichment, the numbers of G418 resistant colonies derived from the infected cells were decreased by half to one-third with the use of the NS cassette (data not shown).

We further investigated whether the enrichment of gene-targeted clones by this NS cassette enables our *PIGA*-based system to detect gene targeting events elicited by plasmid-based targeting vectors. HCT116 and DLD-1 cells were transfected (as a plasmid) with the *PIGA* targeting vector carrying the NS cassette and a promoter-trap system, selected with G418, and then analyzed by fluorescence flow cytometry. This analysis exhibited no increase in the ratio of FLAER-negative cells by the transfection of the above-mentioned *PIGA* targeting vector versus VC (data not shown), again suggesting the difficulty in detecting plasmid-mediated gene targeting in human somatic cell lines using our *PIGA*-based system.

We also designed another NS cassette comprising a splice acceptor site (111 bp) obtained from pIRESneo3 and a SV40 early messenger RNA polyA (238 bp). This newly designed NS cassette was incorporated at the distal end of the 5′ arm in the promoter-trap *PIGA* targeting vector in place of the above-mentioned NS cassette. HCT116 and DLD-1 cells were infected with the resultant AAV-based targeting vector, and the percentages of FLAER-negative cell populations were compared with those of the cells infected with a promoter-trap *PIGA* targeting vector without a NS cassette. This analysis demonstrated no significant difference in FLAER-negative ratios depending on the presence or absence of the newly designed NS cassette (data not shown), suggesting no appreciable capacity of this NS cassette to enrich gene-targeted cell clones.

These experiments investigating small NS cassettes collectively indicate that the introduction of a small NS cassette comprising a splice acceptor and a polyA sites into a promoter-trap targeting vector may moderately elevate the H/R ratio of the targeting vector in human somatic cell lines. However, it is likely that only a limited selection of splice acceptor and polyA sites are capable of elevating H/R ratio. In addition, this increase of H/R ratio may only be detectable with AAV-mediated and not with plasmid-mediated gene targeting in the experimental setting used in this study.

## Discussion

In the current study, we developed a molecular system that exploits a unique property of the *PIGA* gene to evaluate gene targeting efficiencies in human somatic cell lines. The *PIGA*-based system was capable of monitoring AAV-mediated gene targeting in both promoter-trap and non-promoter-trap systems. In previous studies, exogenously introduced marker genes, including GFP and Neo^R^, have frequently been utilized to measure gene targeting frequencies [6], [35]. However, it is often found that the marker genes driven by strong, ectopic promoters are initially expressed constitutively, but eventually reduce their expression to an undetectable level, probably because of epigenetic changes in promoter regions. In contrast, endogenous marker genes, which are regulated by their native promoters, allow for stable monitoring systems that operate in more physiological settings.

The hypoxanthine phosphoribosyltransferase 1 (*HPRT1*) gene is one of the most commonly used endogenous markers [6], [36], [37]. Owing to its localization on the X chromosome, the abrogation of an *HPRT1* allele in cells bearing a single X chromosome leads to the loss of the HPRT1 protein, which results in resistance to 6-Thioguanine (6-TG) and lethality in hypoxanthine-aminopterin-thymidine (HAT) medium. Thus, cells undergoing targeted disruption of *HPRT1* can be quantified by colony formation assays in the presence of 6-TG, while, those undergoing targeted gene correction of mutant *HPRT1* can be identified by cell culture in HAT medium. Other endogenous genes on the X chromosome, such as *IL2RG*, have also been utilized to determine gene targeting efficiencies by PCR-restriction fragment length polymorphism analyses upon ZFN-mediated gene targeting [38]. Compared with these systems, our *PIGA*-based system permits a more high-throughput and convenient measurement of gene targeting frequencies with the aid of fluorescence flow cytometry. Additionally, the *PIGA*-based system offers an option to assay gene targeting frequencies via colony counting after selection of the cells with aerolysin, a toxin that binds to GPI anchors and produces pore-forming channels in cell membranes [39]. Because *PIGA* is also located on the X chromosome, however, *PIGA*-based system can be applied only to cells that carry single X chromosomes, making the cell lines of female origin and many of aneuploid cell lines ineligible for this reporter system. In addition, endogenous marker genes, including *HPRT1* and *PIGA*, may have spontaneous inactivating gene alterations in a subset of cell populations. It is also possible that genes involved in the same biological pathways with *HPRT1* and *PIGA* are biallelically inactivated by spontaneous genetic or epigenetic changes. These events may constitute a background of the reporter systems.

Using the *PIGA*-based system, we evaluated the capacity of small NS cassettes inserted at the end of a targeting vector to improve the efficiency of AAV-mediated gene targeting with promoter-trap strategy, and found approximately 2-fold enrichment of gene-targeted cell clones with one of the NS cassettes. Thus, although this system needs to be further characterized, the PNS strategy described here will be potentially useful in facilitating AAV-mediated gene targeting in human somatic cell lines. However, the enrichment of gene-targeted clones achieved by our PNS strategy was less effective than anticipated. In plasmid-based targeting vectors, similarly, PNS using large NS marker genes has usually achieved only 2–5-fold enrichments of gene-targeted clones. These modest enrichments have been attributed to damage to the NS cassettes upon the random integration of the targeting vectors [40]. However, this hypothesis may not account for our results with an AAV-based targeting vector, since the terminal erosion of AAV vectors upon random integration does not generally proceed over inverted terminal repeats (ITRs); thus, any components within targeting vectors will remain intact in most cases [41]–[43]. We speculate that the observed modest enrichment in this study may be due to splicing around the NS cassette as experienced in insertion mutagenesis [44]–[47].

A variety of technologies including AAV-mediated gene targeting have been developed to achieve genome editing in human cell lines [35], [38], [48], [49]. Among these, AAV-mediated gene targeting has the virtue of being simple, open, cost-sensitive, and sufficiently effective in conducting gene targeting. Moreover, it does not actively promote DNA double strand breaks, thus likely producing a relatively low frequency of off-target DNA cleavages and resulting promiscuous genetic alterations in the genome. Therefore, AAV-mediated gene targeting remains to be an important alternative to conduct genome editing in human somatic cell lines [5], [20]–[28]. This study has shown that the efficiency of AAV-mediated gene targeting can be monitored with a simple procedure using our *PIGA*-based reporter system. This reporter system will be useful to improve the technology of AAV-mediated gene targeting by testing newly designed targeting vectors, optimizing experimental procedures for gene targeting, and screening for genes and drugs that have impact on gene targeting efficiencies. It may also assist in performing comparative evaluation of different human somatic cell lines and thereby identifying cell lines amenable to gene targeting. Such technical improvements will eventually lead to the development of highly efficient technologies of AAV-mediated gene targeting readily applicable to various human somatic cell lines.

## Materials and Methods

### Vector construction

To construct a promoter-trap *PIGA* targeting vector, 5′ and 3′ homology arms, which are both 1.0 kb in size, were amplified by PCR, digested with XbaI and XhoI, respectively, and inserted into pSEPT [30]. To assemble NS cassettes, a fragment consisting of 236 bp of intron 2 and 59 bp of exon 3 from human *BCL2* [50] was PCR-amplified and digested with SacI and NheI. A fragment of 226 bp from BGH polyA was also PCR-amplified and digested with NheI and MluI. Meanwhile, SacI, NheI and MluI restriction enzyme sites were introduced upstream of the 5′ homology arm in the promoter-trap *PIGA* targeting vector, and were then used to incorporate the splice acceptor and BGH polyA sites produced as described above. For the other NS cassette, a splice acceptor site from pIRESneo3 was PCR-amplified, digested with SacI, and ligated at the distal end of the 5′ homology arm in the promoter-trap *PIGA* targeting vector. The resulting plasmid was cleaved with NheI and MluI, and ligated with a XbaI-MluI fragment from pIRESneo3 which contains SV40 early messenger RNA polyA.

To create a *PIGA* targeting vector in which the CMV promoter drives Neo^R^, the promoter-trap *PIGA* targeting vector was cleaved by BamHI and ClaI to remove the promoter-trap module. Meanwhile, pCIneo (Promega) was first digested with HindIII, and the longer fragment was self-ligated and cleaved with BglII and ClaI. A 1.7 kb fragment containing the CMV promoter, Neo^R^, and a polyA was then isolated and ligated to the BamHI-ClaI site of the cleaved promoter-trap *PIGA* targeting vector.

For the construction of a *PIGA* targeting vector in which human *PGK1* promoter drives Neo^R^, the promoter-trap *PIGA* targeting vector was cleaved by BamHI and HindIII, and the longest fragment containing 5′ and 3′ homology arms was recovered. Meanwhile, human *PGK1* promoter was PCR-amplified, digested with BamHI and HindIII, and then ligated to the above-mentioned 5′ and 3′ homology arms. The resulting plasmid was then cleaved with HindIII and ClaI and ligated to a HindIII-ClaI fragment of pCIneo containing the Neo^R^ gene and a polyA.

Constructs were confirmed by DNA sequencing and then used to produce AAV particles with 293T cells and the AAV helper-free system (Agilent Technologies) as previously described [51]. PCR was carried out with the primers listed in Table S1.

### *PIGA* gene targeting assay

The HCT116, DLD-1, and AsPC-1 cell lines were cultured in Dulbecco's modified Eagle&s medium (DMEM; Wako) supplemented with 5% fetal bovine serum (Biowest) and 1% penicillin/streptomycin (Wako). The infection of cells with the AAV-based targeting vectors was carried out as previously described [51] at an MOI of 1×10^4^ unless otherwise noted. The MOI of AAV was determined by its copy number, which was quantified by real-time PCR using StepOnePlus (Applied Biosystems). Initially, the concentration of the solution of plasmid-based *PIGA* targeting vector was assayed with NanoDrop (NanoDrop Technologies) and used to determine its copy number in reference to its molecular weight. This plasmid prep was then diluted and used as a control in real-time PCR. For antibiotic selection, 0.4 mg/ml and 1 mg/ml of G418 (Invitrogen) were used for HCT116 and DLD-1 cells, respectively. After 1 to 2 weeks of G418 selection in 75 cm^2^ flasks, the bulk population of cells was dissociated and stained with FLAER (Pinewood Scientific Services) as per the manufacturer's instructions. FLAER was used at a final concentration of 5 nM. Flow cytometric analyses were carried out with FACSCanto II (BD Biosciences). Cell sorting was conducted with FACSVantage SE (BD Biosciences).

To compare the AAV- and plasmid-based targeting vectors, cells seeded in 75 cm^2^ flasks at 30% confluency were transfected with 7.5 μg/flask of the plasmid-based promoter-trap *PIGA* targeting vector using TransIT-LT1 (Mirus Bio) as per the manufacturer's instructions, and were then selected with G418. The resulting bulk cells were stained with FLAER and analyzed by flow cytometry as described above. Data were compared with those obtained in parallel through the infection of the AAV-based promoter-trap *PIGA* targeting vector.

### PCR

To confirm the homologous integration of the *PIGA* targeting vector by PCR, genomic DNA (gDNA) was extracted from single cell clones using PureLink Genomic DNA Mini Kit (Invitrogen) and used as templates. PCR was performed with Platinum Taq (Invitrogen), the primers shown in Table S1, and a GeneAmp 9700 thermal cycler (Applied Biosystems).

### Southern blotting

Southern blotting was carried out using gDNA extracted with a standard phenol/chloroform extraction and ethanol precipitation protocol. Five micrograms of gDNA was digested with XbaI, electrophoresed on 0.7% agarose gels, blotted onto Hybond-N+ membranes (GE Healthcare), and probed with an approximately 300-bp internal sequence of Neo^R^. Labeling of the Probe was carried out with AlkPhos Direct Labelling and Detection System with CDP-Star kit (GE Healthcare).

### Cell proliferation assay

Cells derived from HCT116 and DLD-1 parental cell lines were seeded in 96-well tissue culture plates at a density of 1,000 cells and 500 cells per well, respectively. Cell numbers were quantified every other day from Day 1 (the day after seeding) to Day 7 using crystal violet staining. For staining, cells in the plates were first washed with phosphate buffered saline (PBS), fixed with 10% buffered formalin (Sigma–Aldrich), stained with 0.2% crystal violet (Sigma–Aldrich), washed with PBS three times, and air-dried. Crystal violet was then dissolved into 10% acetic acid, and absorbance at wavelength 590 nm was measured by VersaMax Microplate Reader (Molecular Devices). For background subtraction, the averaged absorbance of blank wells (medium only) in a tissue culture plate was subtracted from the measurement of each well in the same plate.

### Statistics

The impact of MOI on gene targeting efficiency was analyzed by a one-way factorial analysis of variance (ANOVA). All the other statistical analyses in this study were carried out with a two-way factorial ANOVA. Intercooled Stata (Stata) was used for the analyses.
